# Biomechanical analysis of sacroiliac joint motion following oblique-pulling manipulation with or without pubic symphysis injury

**DOI:** 10.3389/fbioe.2022.960090

**Published:** 2022-09-20

**Authors:** Jing Li, Yikai Li, Ruiyue Ping, Qing Zhang, Hai-Yun Chen, Dingkun Lin, Ji Qi

**Affiliations:** ^1^ Guangzhou University of Chinese Medicine, Guangzhou, China; ^2^ School of Traditional Chinese Medicine, Southern Medical University, Guangzhou, China; ^3^ Department of Dermatology, Guangdong Provincial Hospital of Chinese Medicine, Guangzhou, China; ^4^ Wang Jing Hospital of China Academy of Chinese Medical Sciences, Beijing, China; ^5^ Department of Orthopedics, Guangdong Provincial Hospital of Chinese Medicine, Guangzhou, China; ^6^ Postdoctoral Research Station, Guangdong Provincial Hospital of Chinese Medicine, Guangzhou, China

**Keywords:** oblique-pulling manipulation, sacroiliac joint, biomechanics, pubic symphysis injury, iliolumbar ligament, 3D photogrammetry

## Abstract

**Background:** Oblique-pulling manipulation has been widely applied in treating sacroiliac joint (SIJ) dysfunction. However, little is known about the biomechanical mechanism of the manipulation. This study aims to analyze the SIJ motion under oblique-pulling manipulation, in comparison with compression and traction loads.

**Methods/Study Design:** A total of six specimens of embalmed human pelvis cadavers were dissected to expose the SIJ and surrounding ligaments. Through a servo-hydraulic testing system, biomechanical tests were performed on the stable pelvis and the unstable pelvis with pubic symphysis injury (PSI). A three-dimensional (3D) photogrammetry system was employed to determine the separation and nutation in three tests: axial compression (test A), axial traction (test B), and oblique-pulling manipulation (test C).

**Results:** After applying the testing loads, the range of nutation was no more than 0.3° (without PSI) and 0.5°(with PSI), separately. Except for test B, a greater nutation was found with PSI (*p* < 0.05). Under both conditions, nutation following test A was significantly greater than that of other tests (*p* < 0.05). SIJ narrowed in test A and separated in tests B and C, where the range of motion did not exceed 0.1 mm (without PSI) or 0.3 mm (with PSI) separately. Under both conditions, the separation of SIJ in test C was not as apparent as the narrowness of SIJ in test A (*p* < 0.05). Compared to SIJ, a more significant increasing displacement was found at the site of the iliolumbar ligament (*p* < 0.05). Nevertheless, when the force was withdrawn in all tests, the range of nutation and separation of SIJ nearly decreased to the origin.

**Conclusion:** Pubic symphysis is essential to restrict SIJ motion, and the oblique-pulling manipulation could cause a weak nutation and separation of SIJ. However, the resulting SIJ motion might be neutralized by regular standing and weight-bearing load. Also, the effect on SIJ seems to disappear at the end of manipulation. Therefore, the stretching and loosening of surrounding ligaments need to be paid more attention to.

## Introduction

The sacroiliac joint (SIJ) consists of two parts: the anterior synovial joint (intra-articular part) and the posterior syndesmosis (extra-articular part) (Gartenberg et al., 2021) (Standring, 2016). The SIJ follows a force closure model, characterized by the bony extensions protruding from both the sacral and iliac articular surfaces into the SIJ, forming a blunted, tight, interlocking structure that provides a high surface friction coefficient. This unique structure, combined with extensive ligamental stability, enables the SIJ to maintain vertical stability without additional forces from surrounding musculature (Vleeming and Schuenke, 2019). The primary stabilizers of the SIJ include the sacroiliac, sacrotuberous, and sacrospinous ligaments (Bertoldo et al., 2021). The thoracolumbar fascia connects the gluteus maximus muscle with the latissimus dorsi muscle and then continues with the deep fascia of the limbs (Vleeming et al., 2012; Stecco et al., 2013). Vleeming demonstrated how this aponeurotic structure anchors itself to other bone, muscular structures, and ligamentous in the sacroiliac region and forms a continuum with the lower limb fascia (Carvalho et al., 2013).

Sacroiliac joint dysfunction (SIJD) typically results from abnormal motion and malalignment of the joint. Abnormal motion includes hypo- or hyper-mobile SIJ. Increased secretion of estrogen and relaxation during pregnancy, as well as fetal growth pressure, may lead to hypermobility of the SIJ (Capobianco et al., 2015). In contrast, a sedentary lifestyle and pelvic fractures can cause joint fixation and hypo-mobility (Gartenberg et al., 2021). Although SIJD has been found to be the primary cause of lower back pain in 15%–40% of patients, it is constantly under diagnosed or overlooked and later under treated (Des Jarlais et al., 2004; Cusi, 2010; Grassi Dde et al., 2011; Graup et al., 2014; Buchanan et al., 2021). Standard physical therapy interventions can be employed to correct the underlying pathology and alleviate the symptoms of SIJD. Such interventions include manipulation, mobilization, sacroiliac belts, repetitive exercises, massage, aerobic conditioning, patient education, and electrotherapeutic modalities (Moyer et al., 2004; Huijbregts, 2008). Manipulation is a highly regarded treatment and is known to have a certain therapeutic effect on various diseases, including the treatment of SIJD (Tang et al., 2016). In particular, oblique-pulling manipulation is a simple, effective, and practical method for treating SIJD (Shokri et al., 2018). However, it is still controversial whether it can move the SIJ. Studies have speculated that the mechanism is to stretch and widen the joint space of the SIJ with the help of muscle strength to rotate the SIJ for the purpose of repositioning (Vleeming et al., 1990). Meanwhile, other studies support the opposite idea that the manipulation cannot move the SIJ as the joint is very stable. In addition, pubic symphysis plays an important role in maintaining the stability of SIJ, and the effects of oblique-pulling manipulation on SIJ with or without pubic symphysis injury (PSI) remain unknown.

Therefore, this study aimed to explore the SIJ motion under oblique-pulling manipulation compared to compression and traction loads, *via* biomechanical tests on adult cadaveric specimens.

## Materials and methods

### Ethics

All procedures performed in this study involving human participants followed the Declaration of Helsinki (as revised in 2013). The procedures were approved by the Ethics Committee of Guangdong Provincial Hospital of Chinese Medicine (BM 2022–059). The donors have dedicated their bodies for educational and research purposes to the local Institute of Anatomy prior to death, in compliance with local institutional and legislative requirements.

### Materials

A total of six formalin-embalmed adult cadaveric pelvises were visually selected and examined by X-ray to rule out bone abnormalities such as tumor, fracture, dislocation, deformity, and severe osteoporosis. The three female and three male donors had a mean age of 42 years (range, 35–56 years). The specimens were approved and kept by the Department of Anatomy, Southern Medical University.

### Specimen processing

**FIGURE 1:**
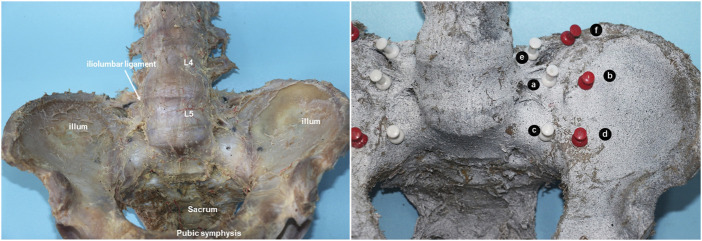
Prepared pelvic cadaveric specimen and the three pairs of markers. **(A)** Medial point in the left and upper sacroiliac joint; **(B)** later point in the left and upper sacroiliac joint; **(C)** medial point in the left and lower sacroiliac joint; **(D)** lateral point in the left and lower sacroiliac joint; **(E)** left transverse process of the fifth lumbar vertebrae; **(F)** attaching point of the left iliolumbar ligament above the iliac crest.

Dissection was performed to expose the sacroiliac joint with intact ligaments, capsule, and pubic symphysis. The specimens were labeled after surface treatment with the imaging agent. Markers were placed on all of the pelvises as follows: two markers were aligned on the medial side of the SIJ and spaced 2 cm apart at a distance of 1 cm from the joint line; two markers were aligned on the lateral side of the SIJ and spaced 2 cm apart at a distance of 1 cm from the joint line. Another two markers were placed on the attachment point of the iliolumbar ligament (IL) on the iliac crest and the costal process of the fifth lumbar vertebra separately. The aforementioned marking points were fixed by marking nails (Figure 1). In addition, the unstable condition with PSI was simulated by sectioning off the pubic symphysis.

### Biomechanical protocol

All biomechanical tests were routinely conducted using the servo-hydraulic material testing system (Bose Electro Force 3520-AT; Bose, MN, United States). Also, the Win Test Digital software was used to control the applied load. During the experimental process, the room temperature was maintained at 20–25°C. To minimize the viscoelastic effect of the specimens, a small-scale loading/unloading pre-treatment of the specimens was performed prior to the experiment, as the previous study described (Wang et al., 2018). In turn, three kinds of biomechanical tests were performed on specimens under stable (SIJ without PSI) and unstable conditions (SIJ with PSI). Each test consisted of two sequential phases (loading phase and unloading phase), as follows:

- *Test A (compression)*: Once each pelvis was fixed to the machine, a progressive compression load of 300 N was applied at a rate of 20 mm/min to each specimen over the lumbar spine (L5) and the sacrum in the axial direction so as to simulate the equivalent of half of the weight of a 60-kg person (standing posture). Then the compression load was unloaded instantaneously.
- *Test B (traction)*: A progressive tensile load of 300 N was applied to each specimen at a rate of 20 mm/min in the axial direction. Then the tensile load was unloaded instantaneously.
- *Test C (oblique-pulling) (rotate to the left)*: In terms of clinical practice and related study, oblique-pulling manipulation was simulated as rotation and traction loading. First, the angle control mode was adopted with a pre-loading angle of 5° at a speed of 1°/sec. Then the maximum loading angle was 10° at a speed of 10°/sec. At the same time, 300 N was uploaded to simulate traction load along with rotation during the manipulation process. Finally, the rotation load was unloaded at the speed of 1°/sec until 0.

**FIGURE 2:**
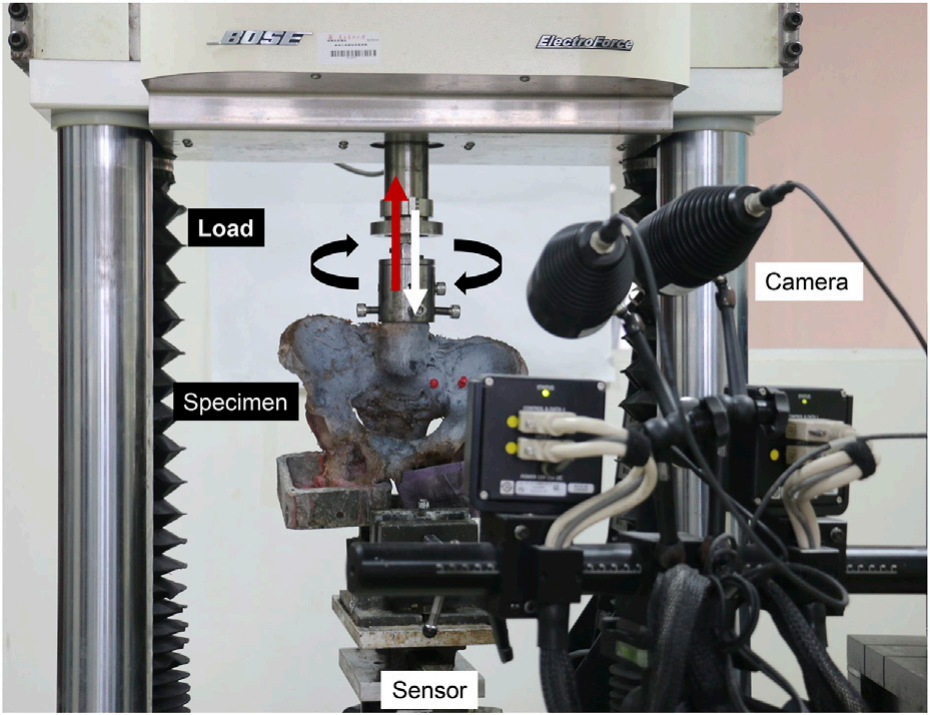
Process of biomechanical tests and the simultaneous noncontact optical 3D strain measurement.

To record the separation and nutation of SIJ during the tests, a noncontact optical 3D strain measuring system (Aramis 3D camera 6 M, GOM, Braunschweig, Germany) was used to visualize the markers. For data acquisition, a frequency of four images/second was used. Through digital photogrammetry, the positions of these markers were calculated with a resolution of 2,448 × 2050 pixels and a precision error of 0.001 mm (Figure 2).

### Mechanical data analysis

To reflect the separation of SIJ, the relative displacements during the loading phase were represented by *L*_ab_, *L*_cd_, and *L*_ef_, respectively. Meanwhile, *L*_ba_, *L*_dc_, and *L*_fe_ represented the relative displacements during the unloading process, respectively. Detailed calculations were as follows: *L*_ab_ = a_1_b_1_−a_0_b_0_, *L*_cd_ = c_1_d_1_−c_0_d_0_, *L*_ef_ = e_1_f_1_−e_0_f_0_, *L*_ba_ = a_2_b_2_−a_0_b_0_, *L*_dc_ = c_2_d_2_−c_0_d_0_, and *L*_fe_ = e_2_f_2_−e_0_f_0_, where a_0_b_0_, c_0_d_0_, and e_0_f_0_ are the initial (no loading) distances between three pairs of markers; a_1_b_1_, c_1_d_1_, and e_1_f_1_ are the distances at the end of loading phase; and a_2_b_2_, c_2_d_2_, and e_2_f_2_ are the distances at the end of unloading phase. In addition, SIJ nutation was determined as the iliac rotations with respect to the sacrum.

**FIGURE 3:**
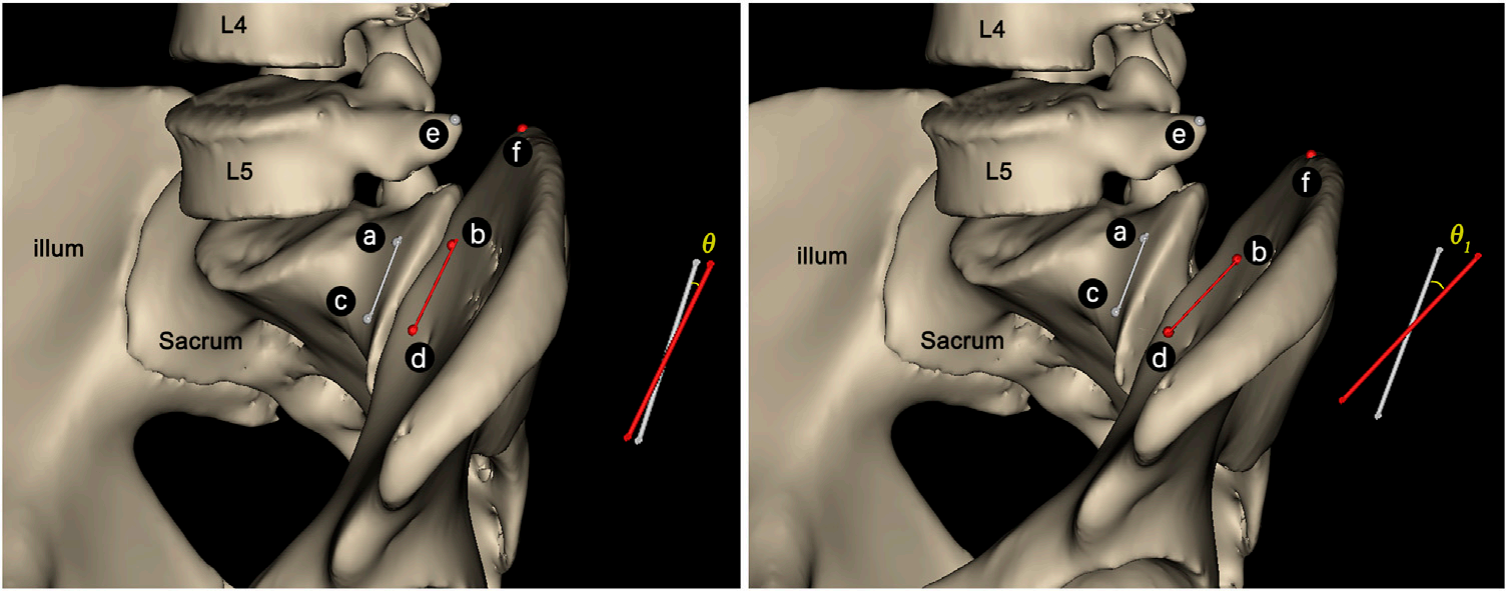
Sketch of *θ* angle between the line **(A,C)** and line **(B,D)**. The angle would increase to *θ*_1_ along with the SIJ nutation **(C)**.

Therefore, the angle between the line ac (from point a to point c) and line bd (from point b to point d) was defined as *θ* (Figure 3). The relative angular displacements during the loading phase (*Δθ*_1_) and unloading phase (*Δθ*_2_) were calculated as follows: *Δθ*_1_ = *θ*_1_−*θ*_0_, *Δθ*_2_ = *θ*_2_−*θ*_0_, where *θ*_0_ is the initial angle, and *θ*_1_ and *θ*_2_ is the angle at the end of the loading phase, unloading phase, respectively.

### Statistical analysis

Variables of the displacement and angle were expressed as mean ± standard deviation. ANOVA of random block design data was used to compare the differences in SIJ relative displacement and angular displacement under different loading conditions. A LSD-t test was used for multiple comparison between groups. An analysis was performed by SPSS software (version 20, IBM Corp). All statistical tests were two-sided, and the level of statistical significance was set at two-sided *p* < 0.05.

## Results

### Overall strain

**FIGURE 4:**
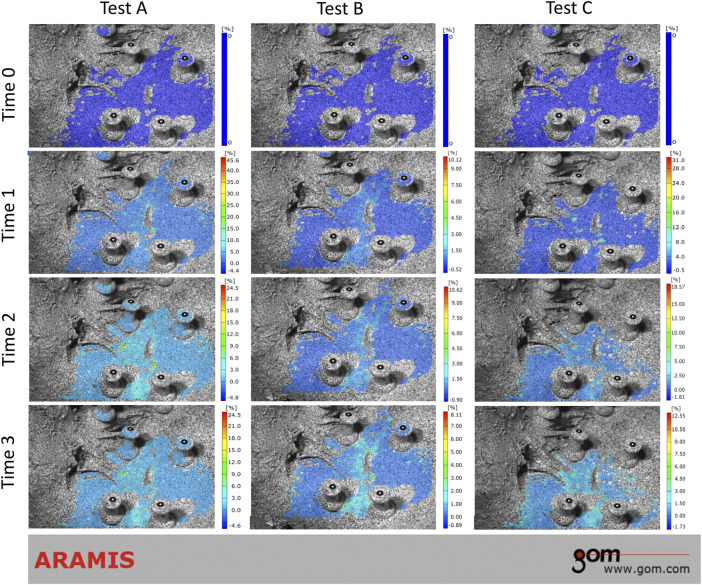
Strain trend of stable sacroiliac joint under three tests (loading phase). Time 0 showed the original condition without any load. Time 1 and 2 showed the process of loading. Time 3 showed the ending condition with loads.

**FIGURE 5:**
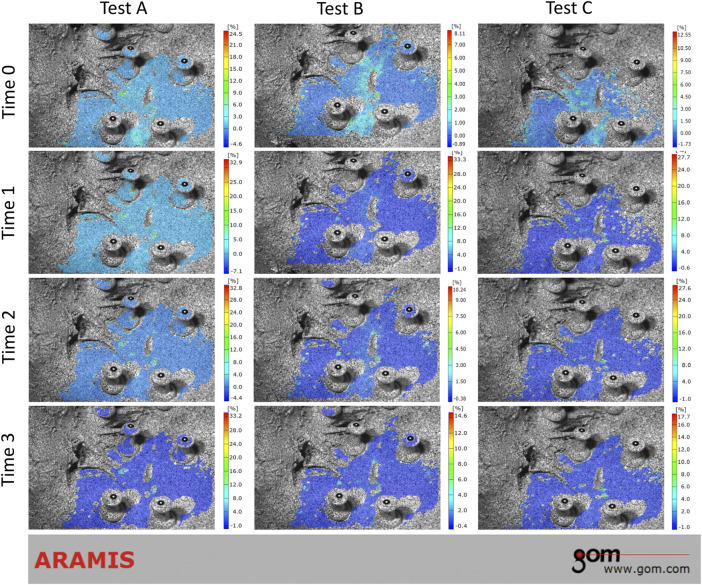
Strain trend of stable sacroiliac joint under three tests (unloading phase). Time 0 showed the original condition with the maximum load, which was the same as the ending condition during loading phase. Time 1 and 2 showed the process of unloading. Time 3 showed the ending condition without any load.

**FIGURE 6:**
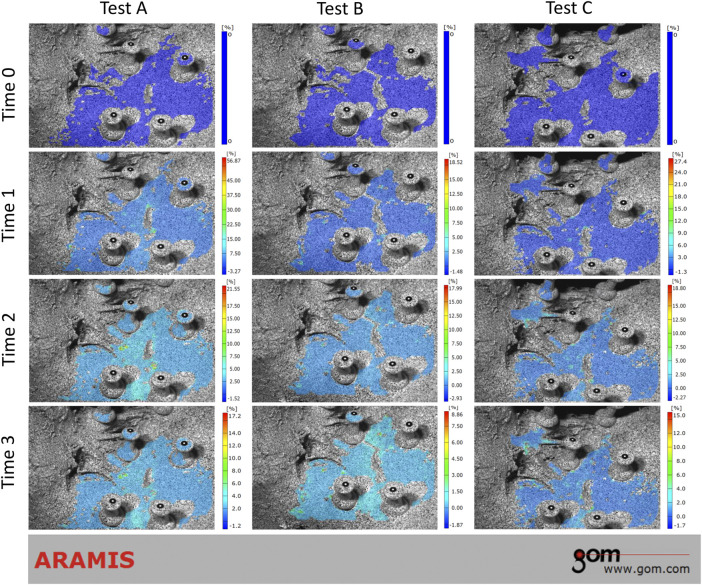
Strain trend of the unstable sacroiliac joint with injured pubic symphysis under three tests (loading phase). Time 0 showed the original condition without any load. Time 1 and 2 showed the process of loading. Time 3 showed the ending condition with loads.

**FIGURE 7:**
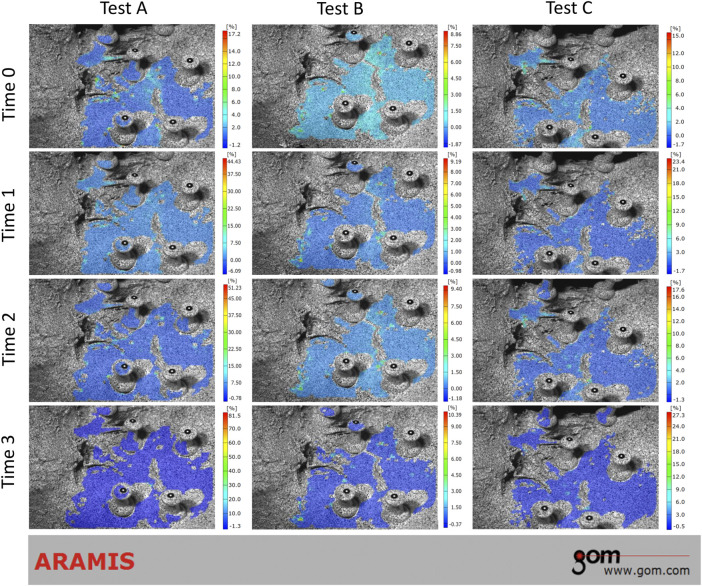
Strain trend of the unstable sacroiliac joint with injured pubic symphysis under three tests (unloading phase). Time 0 showed the original condition with the maximum load, which was the same as the ending condition during loading phase. Time 1 and 2 showed the process of unloading. Time 3 showed the ending condition without any load.

During the loading phase, the uneven strain of pelvic specimens with stable SIJ became more and more evident over time, under three tests. The strain mainly concentrated upon the position of SIJ (Figure 4). In the contrast, during the unloading phase, the strain recovered to the origin state gradually (Figure 5). Despite the strain of pelvic specimen with unstable SIJ showing the same trend, the overall strain around SIJ seemed to be greater than the stable (Figures 6, 7).

### Angular displacement of sacroiliac joint

**FIGURE 8:**
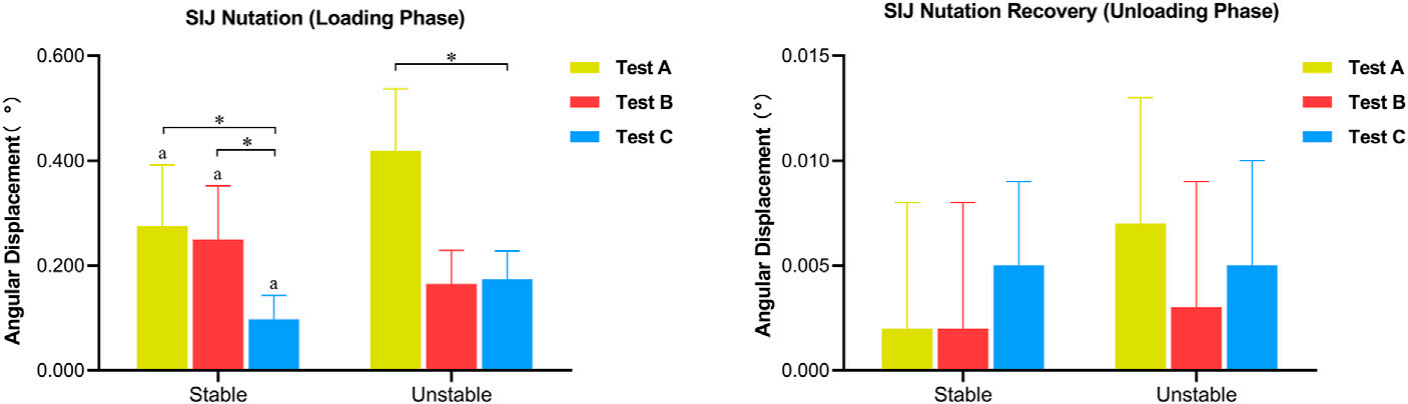
Differences in angular displacement of SIJ (^a^ *P* < 0.05 vs. unstable SIJ; *p < 0.05).

During the loading phase, *θ* in three tests increased gradually, both on the stable and unstable SIJ. For the stable SIJ, the average Δ*θ*_1_ produced by tests A, B, and C was 0.275°, 0.250°, and 0.098°, respectively. For the unstable SIJ, the Δ*θ*_1_ produced by tests A and C were significantly greater than the stable condition (*p* < 0.05), with the average of 0.419° and 0.174°, respectively. However, the average Δ*θ*_1_ produced by test B on the unstable SIJ was 0.165°, which was significantly lower than the stable condition. There was a significant difference in *Δθ*_1_ among three tests either (*p* < 0.05). For both stable and unstable SIJ conditions, *Δθ*_1_ produced by test A was significantly greater than that of the other two tests (*p* < 0.05). *Δθ*_1_ produced by test B was significantly greater than that of test C on stable SIJ (*p* < 0.05), without a significant difference on unstable SIJ (*p* > 0.05) (Table 1; Figure 8).

**TABLE 1:** SIJ nutation and separation during the loading phase.

		N	Angular displacement *Δθ*_1_ (°)	Displacement				
				*L*_ab_ (mm)	*L*_cd_ (mm)	*L*_ef_ (mm)	*F* value	*p* value
Stable SIJ	Test A	6	0.275 ± 0.117^a,d^	− 0.107 ± 0.048^,b,c^	− 0.087 ± 0.025^b,c^	− 0.186 ± 0.049	9.192	0.002
	Test B	6	0.250 ± 0.102^d^	0.036 ± 0.024^b^	0.013 ± 0.007^b,d^	0.186 ± 0.036^a^	82.791	0.000
	Test C	6	0.098 ± 0.045^a^	0.062 ± 0.043^ab^	0.096 ± 0.027^b^	0.220 ± 0.035	33.039	0.000
	*F* value		6.384	4.861	26.671	1.456	N/A	N/A
	*p* value		0.010*	0.024*	0.000*	0.264	N/A	N/A
Unstable SIJ	Test A	6	0.419 ± 0.118^c,d^	− 0.100 ± 0.072^b,c^	− 0.098 ± 0.066^b,c^	− 0.216 ± 0.044	7.146	0.007
	Test B	6	0.165 ± 0.064	0.033 ± 0.019^b,d^	0.016 ± 0.006^b,d^	0.183 ± 0.037^d^	86.926	0.000
	Test C	6	0.174 ± 0.054	0.101 ± 0.041^b^	0.078 ± 0.025^b^	0.259 ± 0.044	41.525	0.000
	*F* value		17.986	3.855	6.474	4.930	N/A	N/A
	*p* value		0.000*	0.045*	0.009*	0.023*	N/A	N/A

^a^ *P* < 0.05 vs. unstable SIJ; ^b^ *P*<0.05 vs. *L*_*ef*_ ; ^c^ *P*<0.05 vs. Test B; ^d^ *P*<0.05 vs. Test C; *p < 0.05.

During the unloading phase, *θ* in three tests decreased to the original angle gradually. The average *Δθ*_2_ produced by tests A, B, and C was 0.002°, 0.002°, and 0.005° for the stable SIJ and 0.007°, 0.003°, and 0.005°, respectively. No significant difference was found between stable and unstable SIJ (*p* > 0.05), or among three tests (*p* > 0.05) (Table 2; Figure 8).

**TABLE 2:** SIJ nutation and separation recovery during the unloading phase.

		N	Angular displacement *Δθ*_2_ (°)	Displacement				
				*L*_ba_ (mm)	*L*_dc_ (mm)	*L*_fe_ (mm)	*F* value	*p* value
Stable SIJ	Test A	6	0.002 ± 0.006	0.003 ± 0.005	0.003 ± 0.001	0.002 ± 0.002	0.309	0.819
	Test B	6	0.002 ± 0.006	0.003 ± 0.005	0.003 ± 0.001	0.002 ± 0.002	0.436	0.729
	Test C	6	0.005 ± 0.004	0.005 ± 0.002	0.002 ± 0.003	0.002 ± 0.003	1.991	0.148
	*F* value		0.688	0.193	1.199	0.659	N/A	N/A
	*p* value		0.518	0.826	0.329	0.532	N/A	N/A
Unstable SIJ	Test A	6	0.007 ± 0.006	0.006 ± 0.002	0.004 ± 0.004	0.005 ± 0.003	0.522	0.604
	Test B	6	0.003 ± 0.006	0.006 ± 0.002	0.004 ± 0.004	0.002 ± 0.003	2.149	0.151
	Test C	6	0.005 ± 0.005	0.004 ± 0.005	0.003 ± 0.001	0.003 ± 0.001	0.209	0.814
	*F* value		0.819	0.891	0.156	1.520	N/A	N/A
	*p* value		0.460	0.431	0.857	0.250	N/A	N/A

### Displacements of sacroiliac joint

**FIGURE 9:**
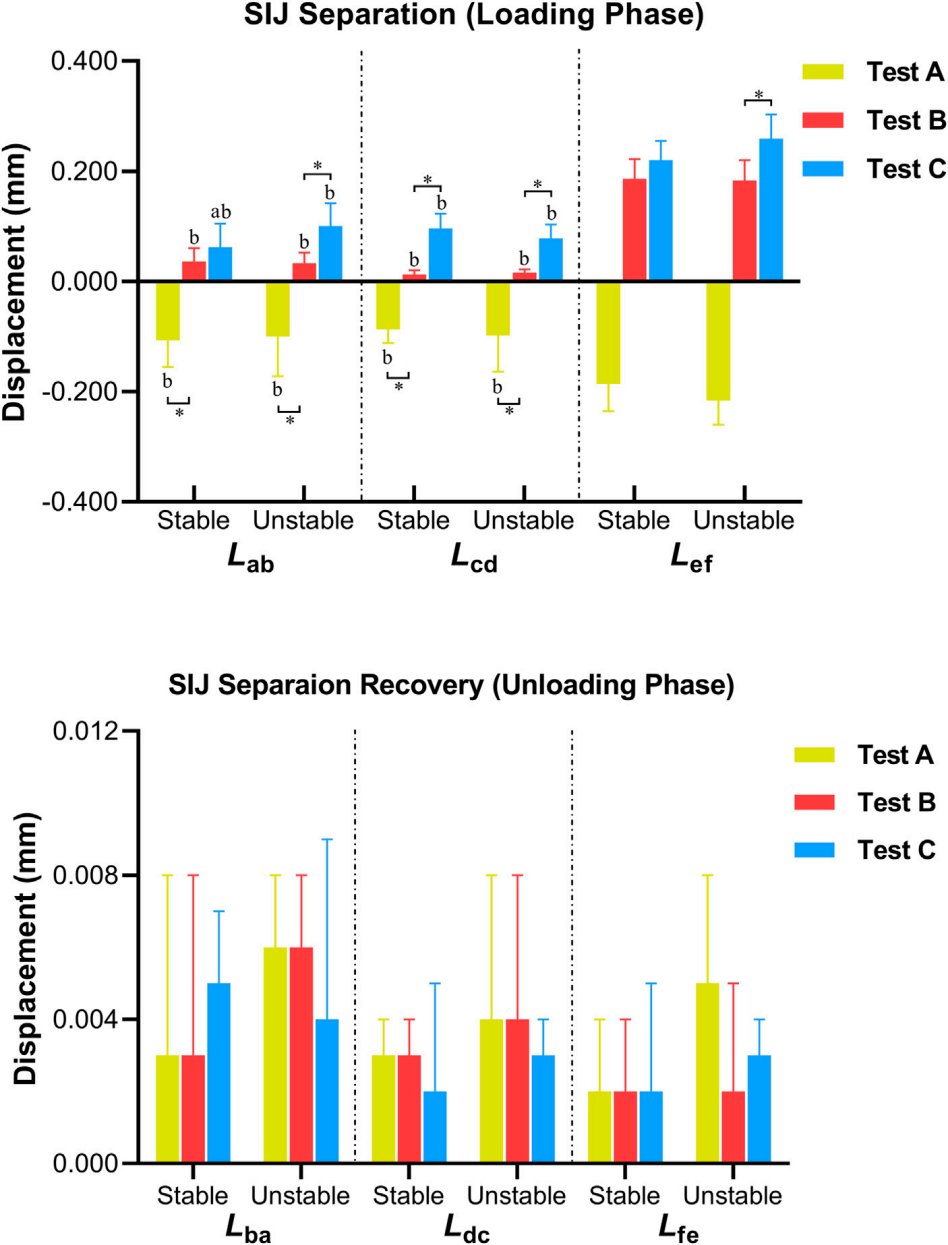
Differences in the displacement of SIJ (^a^ *P* < 0.05 vs. unstable SIJ; ^b^ *P*<0.05 vs. *L*_*ef*_ ; *p < 0.05).

During the loading phase, the distances of ab, cd, and ef in test B and test C increased gradually, both on the stable and unstable SIJ. In the contrast, the aforementioned distances showed a general decreasing trend. For the stable SIJ, *L*_ab_, *L*_cd_, and *L*_ef_ were 0.107 mm 0.087 mm, and 0.186 mm in test A; 0.036 mm, 0.013 mm, and 0.186 mm in test B; and 0.062 mm, 0.096 mm, and 0.220 mm in test C, respectively. No matter in which test, *L*_ef_ was significantly greater than *L*_ab_ and *L*_cd_ (*p* < 0.05). In the comparison among three tests, significant differences were found in *L*_ab_ and *L*_cd_ (*p* < 0.05), without *L*_ef_ (*p* > 0.05). In test A, *L*_ab_ and *L*_cd_ were significantly greater than test B (*p* < 0.05). In test B, *L*_cd_ was lower than test C (*p* < 0.05). For the unstable SIJ, *L*_ab_, *L*_cd_, and *L*_ef_ were 0.100 mm, 0.098 mm, and 0.216 mm in test A; 0.033 mm, 0.016 mm, and 0.183 mm in test B; 0.101 mm, 0.078 mm, and 0.259 mm in test C, respectively. In the three tests, *L*_ef_ was significantly greater than *L*_ab_ and *L*_cd_ (*p* < 0.05). In the comparison among three tests, significant differences were found in *L*_ab_, *L*_cd_, and *L*_ef_ (*p* < 0.05). In test A, *L*_ab_ and *L*_cd_ were significantly greater than test B (*p* < 0.05). In test B, *L*_ab_, *L*_cd_, and *L*_ef_ were lower than test C (*p* < 0.05). Moreover, compared to the stable SIJ, *L*_ab_ in test C was greater (*p* < 0.05); however, no significant difference was found in other displacements (*p* > 0.05) (Table 1; Figure 9).

During the unloading phase, the variation in distances showed the opposite trend. For the stable SIJ, *L*_ba_, *L*_dc_, and *L*_fe_ were 0.003 mm, 0.003 mm, and 0.002 mm in test A; 0.003 mm, 0.003 mm, and 0.002 mm in test B; 0.005 mm, 0.002 mm, and 0.002 mm in test C, respectively. For the unstable SIJ, *L*_ba_, *L*_dc_, and *L*_fe_ were 0.006 mm, 0.004 mm, and 0.005 mm in test A; 0.006 mm, 0.004 mm, and 0.002 mm in test B; 0.004 mm, 0.003 mm, and 0.003 mm in test C, respectively. No significant difference was found among three tests, or between the stable and the unstable SIJs (*p* > 0.05) (Table 2; Figure 9).

## Discussion

The purpose of this study was to evaluate the current evidence on the magnitude of SIJ motion followed by oblique-pulling manipulation. In this study, the relative motion of the SIJ was observed by simulating the compression, traction, and oblique-pulling loads on human pelvic cadaveric specimens. The results revealed that the oblique-pulling manipulation could only cause a slight nutation of the stable SIJ. The relative angular displacement was as small as less than 0.2°, which was significantly less than the angular displacement under axial compression and traction loads. In the meantime, this study found that both the tensile and oblique-pulling loads widened the joint space of the normal SIJ, whereas the compression load narrowed the joint space of SIJ. The phenomenon is consistent with the anatomical features of SIJ. However, the widened joint space caused by the oblique-pulling load was not more than 0.2 mm, indicating that the oblique-pulling manipulation may separate SIJ to a very slight degree. Also, the differences in displacements proved that the oblique-pulling manipulation might have a better effect on SIJ in separation compared to traction therapy. Despite this, no significant difference was found, compared to the physiological compression load. That is, the separation of SIJ caused by oblique-pulling manipulation may recover due to the weight-bearing of the pelvis in the standing and sitting positions. Previously, it has been reported that the particularity of SIJ is that the range of motion (ROM) of the joint is minimal (Le Huec et al., 2019). The SIJ motion has been evaluated using different techniques, such as Doppler techniques, radio stereometric, ultrasound, and Roentgen stereophotogrammetric (Sturesson et al., 1989; Vlaanderen et al., 2005).

Studies have shown that ROM of the SIJ has a maximum rotation of about 1.5° in the axial direction, with 1.2° in males and 2.8° in females (Brunner et al., 1991; Kiapour et al., 2020), and a lateral bend of approximately 0.8° (Miller et al., 1987; Cardwell et al., 2021), and the rotation in different planes can be as small as 0.01°, not more than 3°(Sturesson et al., 1989; Sturesson et al., 2000; Foley and Buschbacher, 2006; Cho and Kwak, 2021). The minimum rotation angle is similar to the minimum rotation angle in this study (0.098°). Some cadaveric studies have shown that the average rotation of the sacrum around the *x*-axis is 3.2° (flexion + extension); fixation of only one iliac bone results in an average rotation of 6.2° (Miller et al., 1987; Sturesson et al., 2000). The angular motion of the SIJ has also been observed by sensors and computer techniques and found to be 9.0 ± 6.5° in the sagittal plane and 5.0 ± 3.9° in the transverse plane (Smidt et al., 1995). The level of motion in patients with SIJ pain never exceeds 1.6 mm in healthy individuals flattened 0.7 mm (Sturesson et al., 1989; H A C Jacob and Kissling, 1995), and the SIJ did not flatten more than 2 mm along the axis (Zheng et al., 1997; Kiapour et al., 2020). Therefore, most current studies conclude that the angular motion of the SIJ generally does not exceed 3°, with a displacement range of 0.3–7 mm (Walker, 1992). The angular displacement of the stable SIJ in this study ranged from 0.098° to 0.275°, with a translation range of 0.013–0.220 mm. Only if the pubic symphysis diastasis occurred to simulate the unstable SIJ, ROM would increase with an angular range of 0.174°–0.419°, as well as a translation range of 0.016–0.259 mm. This finding proved the vital importance of pubic symphysis on the stability or mobility of SIJ again, as related studies have highlighted (Hammer et al., 2019; Ricci et al., 2020). Under this condition, it may become uneasy to realize the mobilization of SIJ with intact pubic symphysis, through oblique-pulling manipulation. Moreover, it has been reported that the mobility of the SIJ depends on a position of the joint and the load it is applied to. Increasing the load on the SIJ leads to a ventral inclination of the sacrum with stretching of dorsal ligaments, which shifts the bone position and interferes with its mobility (Tullberg et al., 1998) (de Toledo et al., 2020). Regarding the clinical procedure, oblique-pulling manipulation resembles high-velocity and low-amplitude thrust manipulation. As demonstrated in related studies, through the Roentgen stereophotogrammetric analysis, high-velocity, and low-amplitude thrust manipulation in the SIJ does not alter the position relationship between the sacrum and the ilium bone in healthy individuals (de Toledo et al., 2020). Although the analyzing method is different, the aforementioned results are consistent with our findings. Therefore, the mechanism of the oblique-pulling manipulation in treating SIJ dysfunction needs to be reconsidered.

Because of its importance in maintaining the joint mobility, ligament around SIJ has aroused far more concern, such as IL, sacrotuberous ligament (STL), and long dorsal sacroiliac ligament (LDL) (Vleeming et al., 1996) (van Wingerden et al., 1993) (de Toledo et al., 2020). STL showed extensive connections with the gluteus maximus muscle, long head of the biceps femoris muscle, and sacrospinous ligament but also has extensive connections to the iliococcygeus muscle on the anterior. It was shown that the incremental load of STL restricts the amount of nutation in the SIJ. LDL is closely related to the aponeurosis of the erector spinae muscle and the posterior layer of the thoracolumbar fascia (van Wingerden et al., 1993). Counternutation in the SIJ increases tension of the LDL, and the nutation slackens it. Therefore, the tension applied to the dorsal sacroiliac ligament, or the LDL appears to restrict the contrary movements in the SIJ. Both ligaments are partially connected (Vleeming et al., 1996) (van Wingerden et al., 1993). In this study, during the oblique-pulling, compression, and tensile tests, another important finding was that *L*_ef_ increased more obviously than the relative displacements at sites above and below the SIJ both on the stable and unstable joints. Taking the anatomical location into consideration, we focused on the biomechanical response of IL. IL, as an important primary source of low back pain (Sims and Moorman, 1996), originates from the fourth and fifth lumbar costal process to the iliac crest. Numerous studies have descriptions sketched the shape of IL. For example, Pool-Goudzwaard et al. characterized the ligament up to seven portions, especially including the sacroiliac part. This part originated on the sacrum and blended with the interosseous sacroiliac ligaments, with the length of 30.5 in women or 31.0 mm in men, the width of 12.7 mm in women or 14.5 mm in men, and the thickness of 1.6 mm in women or 1.5 mm in men (Pool-Goudzwaard et al., 2001). In addition, Hammer et al. described the minimal two parts of this ligament, mainly composed of the anterior part and the posterior part, with the length of 25–30 mm and the thickness of 4 mm (Hammer et al., 2010). Owing to the aforementioned characteristics, it has been described that IL restricts the movement of the SIJ, and the effect may be associated with the thickness, the length, and the angle between the insertion of the anterior and posterior parts. In the sagittal plane, the movement of SIJ performed as nutation. Previous biomechanical tests on embalmed pelvic specimens have found that the slope of the SIJ load-nutation curve would increase 28.1%, when the IL was totally cut. That is, injury to the ligament could result in increased up and down movement of the SIJ in sagittal plane, and this should be considered in patients presenting with symptoms related to this hypermobility (Pool-Goudzwaard et al., 2001; Pool-Goudzwaard et al., 2003). Therefore, we speculated that oblique-pulling manipulation might have a better effect of releasing and stretching on IL, and the effect is more apparent than the effects on SIJ itself. Studies have shown that the friction and coupling of the SIJ articular surfaces combined with the surrounding ligaments made the SIJ more stable (Snijders et al., 1993) (Cardwell et al., 2021).

To further explore the possible biomechanical effects of oblique-pulling manipulation on SIJ, in this study, the strain and relative motion of the SIJ were observed by simulating the loading of human pelvic specimens. Positive effects of manipulation may be promoting normal joint mobility, which may release articular or related soft tissue adhesions and synovial folds (Tullberg et al., 1998). There is evidence that manual therapy may refine the SIJ function by acting on nearby muscles (Behdad Hamidi-Ravari et al., 2014). Muscles generate strength, guide movements, and increase pelvic girdle stability (Vleeming et al., 2012). Previous studies on manipulation have lacked in-depth research on the immediate biomechanical effect on the joint when the force was withdrawn. In order to solve this problem, this study did not only observe nutation and separation of SIJ during the loading process but also measured them during the unloading process. The final results showed that the effect disappeared on the normal SIJ, when the force was withdrawn. The nutation and separation recovered nearly after withdrawing the loads, regardless of the stable or unstable joint. Since it was a transient effect, the weak effect on the SIJ produced by the oblique-pulling manipulation may not be sustainable. This is another reason to suspect that promoting joint mobility is not an essential mechanism of oblique-pulling manipulation for the treatment of SIJ dysfunction.

However, it should be mentioned that there are some limitations in the current study. First, formalin-embalmed specimens rather than fresh-frozen specimens were tested, because of financial constraints and considerations about the less ROM of SIJ. The fixation methods may decrease the biomechanical properties of soft tissue and bone may decrease, further resulting in few elongations seen in the tests. Second, no bone density scan was performed before test; thus, the effects of osteoporosis on the biomechanical response remain unknown. Third, the effects of loads on sacroiliac nodal ligament and posterior long sacroiliac ligament were not well investigated by 3D photogrammetry, owing to the complex and irregular posterior structure. Finally, as with other cadaver studies, muscle forces were not simulated. All of the issues need to be further investigated.

## Conclusion

The oblique-pulling manipulation could cause the slight nutation and separation of SIJ; however, the resulting SIJ motion is not more than that of normal people standing and bearing weight. At the same time, the effect of the manipulation is transient, and the effect disappears after the force is removed. Manipulation has a weak effect on the motion of SIJ and thus triggering the SIJ may not be the primary mechanism of the oblique-pulling manipulation. The stretching and loosening of the ligaments around the SIJ by the oblique-pulling manipulation to achieve the therapeutic effect might be another mechanism of action of the manipulation.

## Data Availability

The raw data supporting the conclusions of this article will be made available by the authors, without undue reservation.
